# Case of Displaced Glenoid Fracture After Fall: Subtle Findings with Significant Implications for Trauma Patients

**DOI:** 10.5811/cpcem.2021.2.51053

**Published:** 2021-03-24

**Authors:** Solomon Sebt, Mathias Barden, Eric Leroux

**Affiliations:** Eisenhower Medical Center, Department of Emergency Medicine, Rancho Mirage, California

**Keywords:** Scapula fracture, trauma, glenoid fracture, case report

## Abstract

**Case Presentation:**

A 64-year-old man presented to the emergency department with a chief complaint of left shoulder pain after a mechanical fall from standing. Plain radiography revealed a displaced fracture of the inferior glenoid rim. A computed tomography further characterized the fracture and the patient was taken emergently by an orthopedic surgeon for open reduction and internal fixation.

**Discussion:**

Scapula fractures, especially isolated glenoid rim fractures, are rare and most typical of high-energy mechanism traumas. A missed or delayed diagnosis can result in long-term suffering and disability. Awareness of radiographic as well as physical findings and the subsequent classification system described below can optimize outcomes for trauma patients with glenoid fractures.

## CASE PRESENTATION

**Image 1:**
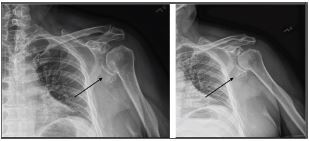
Anteroposterior (left) and oblique (right) views of the left shoulder plain radiograph, showing the inferior rim of the fractured glenoid with extension through the body displaced inferiorly (arrows). Based on the Ideberg classification system of glenoid fractures, this fracture pattern is type II.

**Image 2:**
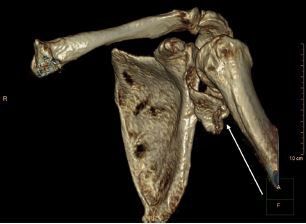
Computed tomography three-dimensional reconstruction of the shoulder showing proper articulation of humeral head within the glenoid fossa at the correct level. Fracture fragment noted by the arrow.

A 64-year-old man presented to the emergency department (ED) complaining of an inability to move his left arm following a mechanical fall during which he landed on his shoulder. On physical exam, the patient was unable to actively abduct, internally or externally rotate the shoulder, and resisted the passive performance of these movements. Radial and ulnar pulses were symmetric and intact. Sensation to light touch was intact throughout the deltoid, forearm, and hand. Symmetric and full strength was observed on wrist flexion and extension, symmetric and full strength with finger adduction and abduction, and no limitation in active extension of the metacarpophalangeal joints. Plain radiography revealed a fracture of the inferior glenoid rim (Image 1). The case was then discussed with the on-call orthopedic surgeon who requested a computed tomography (CT) with three-dimensional (3D) reconstruction (Image 2).

## DISCUSSION

Scapular fractures are rare and commonly missed in patients with high-impact blunt trauma.^4^ The two strongest predictors for missing these fractures are the experience of the emergency physician and the timeliness of orthopedic consultation – earlier intervention associated with better outcomes.^4^ When inappropriately treated, glenoid fractures can cause severe morbidity in all age groups, such as nonunion, severe osteoarthritis, and superior shoulder suspensory complex injury, which plays a key role in shoulder joint stability.^3^

Accurate and expeditious identification and classification of this injury type is critical for optimizing patient outcomes. The Ideberg classification characterizes six glenoid fracture types and is the most commonly used system to describe fractures of the glenoid fossa and rim (Table).^1^,^5^ Our patient’s fracture was classified as type II glenoid fossa fracture with inferior neck extension (Image 1). Typically, fractures within type II or greater require some measure of surgical intervention. Physical exam findings such as positive apprehension, relocation, and hyperabduction tests are highly specific for glenohumeral instability and subsequent glenoid fractures. Plain radiography is the appropriate imaging choice for identifying these glenoid fractures.^4^ The recommended radiographic views for detecting this fracture pattern include true anteroposterior and oblique. Computed tomography (CT) combined with 3D reconstruction is recommended if intra-articular involvement or significant displacement is suspected, as these images permit accurate classification of the fracture and inform subsequent surgical decisions (Image 2).^1^ The American Osteopathic Foundation also offers a widely used classification system for glenoid fractures: F0 = fracture of the articular segment, not involving the glenoid fossa; F1 = simple glenoid fossa fractures; and F2 = multi-fragmentary glenoid fossa fractures.^1^ The Ideberg classification is the system preferred by the orthopedic community as it is more descriptive in its characterization of fracture patterns.

**CPC-EM Capsule**

What do we already know about this clinical entity?

*Scapular fractures are rare and commonly missed in patients with high-impact blunt trauma but even more so in the setting of low-velocity trauma.*

What is the major impact of the image(s)?

*If inappropriately treated, glenoid fractures can cause severe morbidity, such as nonunion, severe osteoarthritis, and shoulder joint instability.*

How might this improve emergency medicine practice?

*Recognizing glenoid fractures and ensuring that surgical candidates are promptly identified have significant implications on long-term patient morbidity.*

The patient described above suffered an F1 fracture, for which non-operative treatment is typically indicated, as 90% are stable and heal well with two weeks of sling stabilization followed by early motion.^2^ However, our patient also demonstrated greater than 10 millimeters (mm) of intra-articular displacement and therefore appropriately was offered and underwent surgery. Indications for surgical repair include involvement of greater than 25% of glenoid with subluxation of the humerus, greater than 5 mm of glenoid articular step off, or greater than 10 mm of intra-articular displacement on CT imaging.^3^ However, operative decisions are informed by the entire clinical picture, including the degree of shoulder joint instability and the patient’s lifestyle goals.

Comprehensive care of patients with scapula or shoulder injuries in the ED is facilitated through a combination of a thorough and targeted physical exam, appropriate radiographic studies, and consultation with an orthopedic specialist. Recognizing glenoid fractures and ensuring that surgical candidates are promptly identified have significant implications on long-term patient morbidity.

## Figures and Tables

**Table:** Ideberg classification of glenoid fractures.

Type	Fracture description
Ia	Anterior glenoid rim fracture
Ib	Posterior glenoid rim fracture
II	Glenoid fossa fracture with inferior neck/body extension
III	Glenoid fossa fracture with superior neck/body extension
IV	Glenoid fossa fracture with medial body extension
Va	Glenoid fossa fracture with medial and inferior extension (II+IV)
Vb	Glenoid fossa fracture with medial and superior extension (III+IV)
Vc	Glenoid fossa fracture with medial, inferior, superior extension (II+III+IV)
VI	Glenoid fossa fracture with severe comminution
